# DILS: depth incremental learning strategy

**DOI:** 10.3389/fnbot.2023.1337130

**Published:** 2024-01-08

**Authors:** Yanmei Wang, Zhi Han, Siquan Yu, Shaojie Zhang, Baichen Liu, Huijie Fan

**Affiliations:** ^1^State Key Laboratory of Robotics, Shenyang Institute of Automation, Chinese Academy of Sciences, Shenyang, China; ^2^Institutes for Robotics and Intelligent Manufacturing, Chinese Academy of Sciences, Shenyang, China; ^3^University of Chinese Academy of Sciences, Beijing, China

**Keywords:** training strategy, network prior, depth incremental learning, local supervision, knowledge transfer

## Abstract

There exist various methods for transferring knowledge between neural networks, such as parameter transfer, feature sharing, and knowledge distillation. However, these methods are typically applied when transferring knowledge between networks of equal size or from larger networks to smaller ones. Currently, there is a lack of methods for transferring knowledge from shallower networks to deeper ones, which is crucial in real-world scenarios such as system upgrades where network size increases for better performance. End-to-end training is the commonly used method for network training. However, in this training strategy, the deeper network cannot inherit the knowledge from the existing shallower network. As a result, not only is the flexibility of the network limited but there is also a significant waste of computing power and time. Therefore, it is imperative to develop new methods that enable the transfer of knowledge from shallower to deeper networks. To address the aforementioned issue, we propose an depth incremental learning strategy (DILS). It starts from a shallower net and deepens the net gradually by inserting new layers each time until reaching requested performance. We also derive an analytical method and a network approximation method for training new added parameters to guarantee the new deeper net can inherit the knowledge learned by the old shallower net. It enables knowledge transfer from smaller to larger networks and provides good initialization of layers in the larger network to stabilize the performance of large models and accelerate their training process. Its reasonability can be guaranteed by information projection theory and is verified by a series of synthetic and real-data experiments.

## 1 Introduction

Modern deep learning models typically have complex network structures and a large number of parameters, requiring significant computing resources and time for training. Therefore, researchers have begun exploring methods for transferring knowledge from pretrained models to new models in order to speed up the training process and improve their performance. However, current knowledge transfer methods are typically based on networks of the same size (Yang et al., 2023), such as weight sharing, feature transfer, or knowledge transfer from deeper to shallower networks (Huang et al., 2022; Shi et al., 2023), such as knowledge distillation, and network pruning. There is a lack of knowledge transfer strategies from shallower to deeper networks. With the development of large models and frequent updates, network sizes have become increasingly larger. Research on knowledge transfer methods from small to large models is necessary. However, several reasons prevent the realization of such knowledge transfer. One reason for the limitation of knowledge transfer from smaller to larger networks is the inability to initialize redundant layers and parameters in the larger network. Traditional end-to-end training wastes a significant amount of time and computing power, restricts network flexibility, and hinders the inheritance of prior knowledge. We aim to propose a training strategy that enables knowledge transfer from smaller to larger networks and provides good initialization of layers in the larger network to stabilize the performance of large models and accelerate their training process.

**Figure 1:**
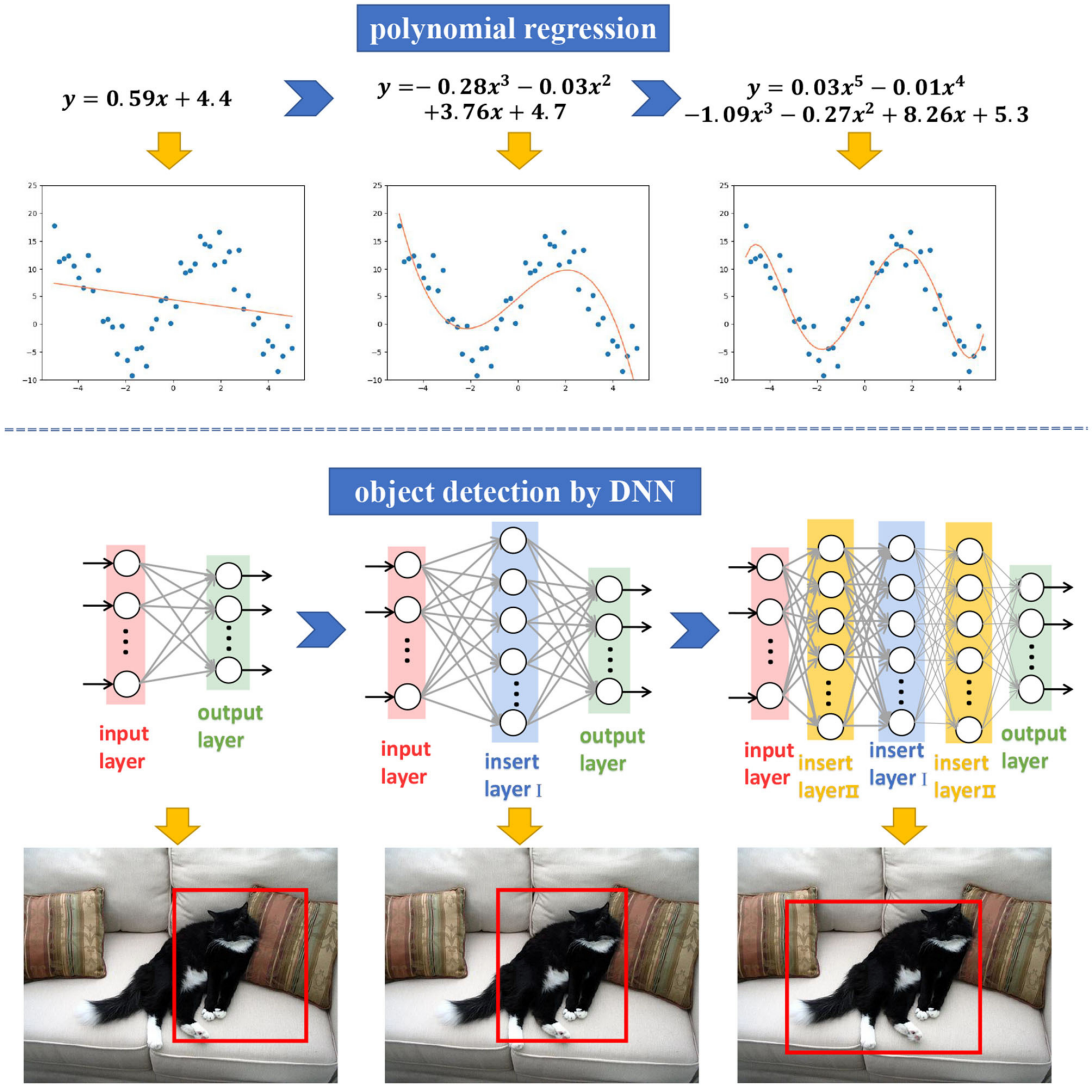
Regression ability gets better along with polynomial degree getting higher. Similarly, performance increases along with network getting deeper.

To solve this issue, we consider an incremental learning strategy. The idea is motivated by the data regression via continuous numerical approximation, e.g., polynomial regression. As shown in Figure 1, by adding the degree of the polynomial, it can increase the regression accuracy for a given set of data. Similarly, as the net deepens, its ability may be enhanced gradually, e.g., more accurate object detection. Similarly, by adding new layers to the network, it can achieve better scalability while inheriting the original performance.

**Figure 2:**
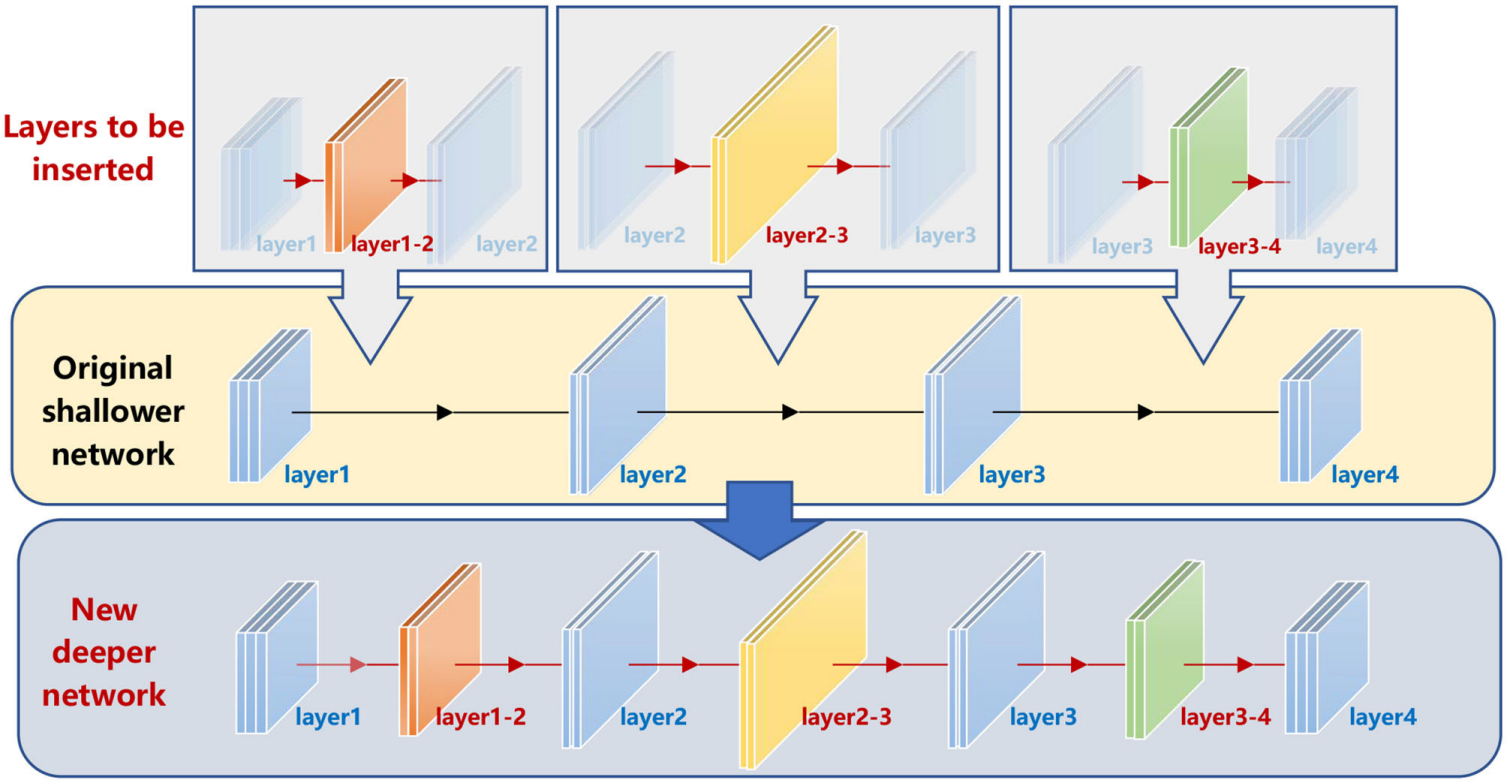
Depth incremental learning model. The original shallow net is well-trained. Then, we insert the orange, yellow, and green layers to the original net. In the Stage I training process, the mapping relations of the original net are invariant to realize stability-based supervision. Then, the new deeper net inherits information of the original shallower net. At the same time, with the number of layers increasing, the solution domain of the mapping relations is expanded, so that the stage II plasticily-based superbision is complished.

We name this strategy the depth incremental learning strategy (DILS). The main idea is illustrated in Figure 2. For a given shallower net (e.g., 4-layer), a set of new layers are inserted (colored orange, yellow, and green, respectively), whose connections with the original layers are trained based on the original network. Then, a new deeper net (7-layer) is obtained, which realizes a flexible ability increment. By repeatedly performing such operations, the net depth increases gradually and achieves knowledge transfer from a small network to a large network.

There are three benefits of the proposed DILS. First, it realizes a continuous learning sequence. The new deeper net is generated based on the shallower net, which has been already well-trained. Thus, it inherits the learned knowledge from the shallower net. Consequently, it gives the new net a good “initialization” for further training. Second, it saves plenty of computing resources by deepening the network gradually to find the best fitting depth of the network. It starts from a shallow net, which is easier to train. The subsequent training for the inserted layers each time is also very easy and time-saving because the new layers are well-initialized based on the original net. Furthermore, it is very flexible for both learning procedure and actual implementation. In other words, the training procedure can stop at any depth to meet the required accuracy. Imagining the following scenario, at the beginning, we want to deploy a network on a light device of lower computing power and do not demand very high task accuracy. Then, a shallow net is enough and suitable. But later, we update the device with higher computing power and become caring more about the accuracy. We can just deepen the previous network to increase the performance within very short training time.

The reasonability and effectiveness of DILS can be explained in both biological systems and computational models studies, and it is well-admitted that an efficient training procedure should make a good balance between stability and plasticity (Ditzler et al., 2015). The mechanisms of neurosynaptic plasticity regulate the stability–plasticity balance in multiple brain areas (Douglas et al., 1995). On the basis of shallow network prior, local stability supervision ensures the performance of the network, while global plasticity supervision enhances the network's efficiency.

It can also be interpreted as an information projection procedure (Zhu and Mumford, 1997; Zhu et al., 2010; Si and Zhu, 2011). From the statistical aspect, the goal of a deep net is to learn a distribution *p* to approximate the distribution *q* of a given task. DILS tries to find a sequence of models *p*_*i*_, which can gradually approach *q*, as shown in Equation (1):


(1) $$\begin{array}{l} p_{1}\to p_{2}\to \cdots \to p_{k}\;to\;q, \end{array}$$

in terms of minimizing the Kullback-Lebler divergence *KL*(*q*∥*p*). For each step, the incremented net is pursued by the following optimization problem, as shown in Equation (2):


(2) $$\begin{array}{l} p_{i}^{*}=\arg\min KL\left(p_{i}∥p_{i-1}\right). \end{array}$$

To sum up, the main contributions of this study are as follows:

- We propose a depth incremental learning strategy (DILS), which enables knowledge transfer from smaller to larger networks and provides good initialization of layers in the larger network to stabilize the performance of large models and accelerate their training process.
- The methods for optimizing the new added parameters along with the new layers are proposed, which guarantees the new deeper net inherits the knowledge learned by the original shallower net.
- DILS enhances the efficiency and flexibility of deep network training, which is verified by a series of synthetic and real-data experiments.

## 2 Related studies

In this section, knowledge transfer methods between networks are initially introduced, followed by a discussion of the more widely researched knowledge transfer method from large networks to small networks, known as network compression.

### 2.1 Knowledge transfer between networks

The common methods for knowledge transfer between networks include fine-tuning, feature extraction, model distillation, and pruning. Fine-tuning (Yosinski et al., 2014) is a knowledge transfer method that involves using a pretrained model as the initial parameters and continuing training on a new task. During the fine-tuning process, the learning rate is usually decreased to avoid disrupting the weights of the pretrained model, and data augmentation is applied to the new dataset to prevent overfitting. Fine-tuning has become one of the most common knowledge transfer methods in computer vision. Feature extraction (Chatfield et al., 2014) is a knowledge transfer method that involves using a pretrained model to extract features, and then passing these features to a new model for classification. Typically, the convolutional layers of a pretrained model are used as the feature extractor, and the features are then passed to fully connected layers for classification. This method is usually more stable than fine-tuning and requires fewer computing resources. Model distillation (Hinton et al., 2015) is a knowledge transfer method that involves using a larger model (usually called the “teacher model”) to “teach” a smaller model (usually called the “student model”). During the training process, the student model attempts to replicate the predictions of the teacher model in order to transfer its knowledge. This method can reduce the size of the model, thereby improving its efficiency (Lan et al., 2022; Hu et al., 2023). Model pruning (Han et al., 2015) is a technique of compressing the model's size by removing unimportant neurons or connections. One can use pruning on a pretrained model, followed by fine-tuning or feature extraction to transfer knowledge, whereas fine-tuning and feature extraction are mostly based on networks of the same size, and knowledge distillation and pruning involve transferring knowledge from large to small networks, our method achieves knowledge transfer from small to large networks.

### 2.2 Neural architecture searching methods

Neural architecture searching (NAS) aims at designing an appropriate neural architecture that achieves the best possible performance. NAS implemented by reinforcement learning (RL) methods has reached state-of-the-art accuracy results on image classification tasks. This demonstrates that automated neural architecture design is feasible (Baker et al., 2016; Jaafra et al., 2019). However, NAS consumes a large amount of computing resources, which is catastrophic for reproduction (Zela et al., 2020) and further research (Pham et al., 2018; Bashivan et al., 2019; Zheng et al., 2019). Even more unfortunately, it is extremely task-specific and lacks of plasticity. For even a small change to the original task, the learned architecture may exhibit poor performance, and it has to search a new neural architecture to solve the slightly altered task. In comparison, DILS is very plastic by gradually incrementally inheriting and learning a series of networks of different depths that can deal with this situation flexibly. More importantly, DILS can realize knowledge inheritance from small structure networks to large ones.

### 2.3 Network compression methods

Network compression methods are developed to speed up computing and save storage space. Among them, low-rank decomposition-based approaches (Oseledets, 2011; Kim et al., 2015; Zhao et al., 2016) conduct decomposition on convolutional filters that are viewed as matrices or tensors. Pruning-based approaches (Wen et al., 2016; Li et al., 2020) compress the network by deleting relatively unimportant weights on different levels, including the filter, channel, and layer levels. Knowledge distillation based approaches (Choi et al., 2022; He et al., 2022) extract knowledge from the teacher network and derive a more compact student network.

Although the network compression method can realize a more lightweighted architecture. However, the compression procedure is also time-consuming and sometimes difficult to calculate. In comparison, DILS starts by training a shallow network, which saves plenty of computing resources. Furthermore, it is very flexible to grow to an appropriate depth to meet the required accuracy. Network compression facilitates the transfer of knowledge from larger networks to smaller networks. Conversely, DILS serves as a complementary technique by enabling knowledge transfer from smaller networks to larger networks.

## 3 Proposed method

### 3.1 Depth incremental learning strategy

Suppose for a target task *y* = *f*(*x*), we already have an *L*-layer deep nets for approximating the function *f*,


(3) $$\begin{array}{l} H_{L}\left(x\right):=h_{\left\{d_{0},\cdots \;,d_{L},\sigma \right\}}\left(x\right)=\vec{a}\cdot {\vec{h}}_{L}\left(x\right), \end{array}$$

(4) $$\begin{array}{l} {\vec{h}}_{k}\left(x\right)=\sigma \left(W_{k}*{\vec{h}}_{k-1}\left(x\right)\right),\;k=1,2,\cdots \;,L, \end{array}$$

in which, *L* ∈ ℕ is the depth of the network, *d*_*k*_ and ${\vec{h}}_{k}\left(x\right)$ are the width and the feature map of the *k*th hidden layer, respectively. $\vec{a}$ is the weight of the output layer. σ(·) is activation function, e.g., Sigmoid, ReLU. “*” here represents the operation between features and input feature maps, which can be fully-connect, convolution, etc.

We aim to achieve knowledge transfer from a small network to a large network.


$$H_{L+l}\left(x\right):=h_{\left\{d_{0},\cdots \;,d_{i_{1}},\cdots \;,d_{i_{k}},\cdots \;,d_{i_{l}},\cdots \;,d_{L},\sigma \right\}}\left(x\right)$$

of depth *L* + *l* based on *h*_{_*d*_0__, ⋯ , *d*_*L*_, σ}_, satisfying Equation (5)


(5) $$\begin{array}{l} ∥H_{L+l}\left(x\right)-f\left(x\right)∥<∥H_{L}\left(x\right)-f\left(x\right)∥. \end{array}$$

Here, ∥·∥ measures the approximation error of network *H* to the target function *f*.

**Figure 3:**
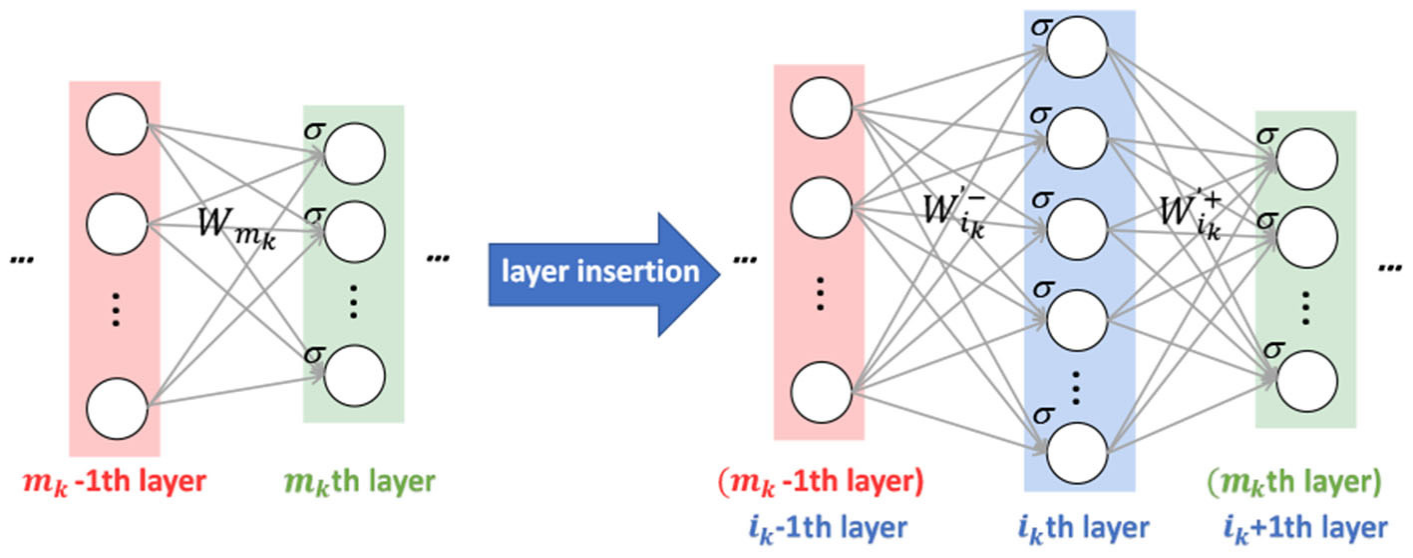
Illustration of stage I. Assume that the original net is with two layers. We add a new layer to the net to boost its expressive capability. To ensure that the newly added layer has good initialization, local stability supervision is employed to make the effect of the inserted net equal to the original net.

For realizing such goal, we provide a two-stage learning strategy:

**Stage I:** Insert new *l* layers (as shown in Figure 3) and initialize the new parameters $W^{\prime }={\left\{W_{i_{k}}^{{\;}^{\prime }-},W_{i_{k}}^{{\;}^{\prime }+}\right\}}_{k=1}^{l}$ based on the old shallower network *H*_*L*_ by solving the following optimization problem as shown in Equation (6)


(6) $$\begin{array}{l} \underset{W^{\prime }}{\min}∥H_{L+l}^{\prime }\left(x\right)-H_{L}\left(x\right)∥. \end{array}$$

For one specific new layer (e.g., the *i*_*k*_th), supposing it is inserted between the (*m*_*k*_ − 1)th and the *m*_*k*_th layer of the old network, the original parameters *W*_*m*_*k*__ (the connection between the (*m*_*k*_ − 1)th and the *m*_*k*_th layer) are replaced by $W_{i_{k}}^{{\;}^{\prime }-}$ and $W_{i_{k}}^{{\;}^{\prime }+}$, which are the new connections of the *i*_*k*_th layer with the (*m*_*k*_−1)th and the *m*_*k*_th layer, respectively.

**Stage II:** Fine-tune the initialized network $H_{L+l}^{\prime }$ by a small set of training data and comparatively less epochs.

The two-stage strategy makes the new network inherit the learned knowledge of old network so that realizes the continuity of incremental learning and make it much easier to train the deeper network.

For better guaranteeing the property of local stability so that further enhancing the training efficiency, we divide the problem (6) into *l* sub-problems as shown in Equation (7),


(7) $$\begin{array}{l} \underset{W_{i_{k}}^{{\;}^{\prime }-},W_{i_{k}}^{{\;}^{\prime }+}}{\min}∥\left({\vec{h^{\prime }}}_{i_{k}+1}\left(x\right)-{\vec{h}}_{m_{k}}\left(x\right)\right){|}_{{\vec{h}}_{m_{k}-1}\left(x\right)}∥,k=1\ldots l. \end{array}$$

In the above optimization problem, we only consider the local network around the *i*_*k*_th layer of the new network. The goal is to reproduce the mapping relationship from the (*m*_*k*_−1)th layer to the *m*_*k*_th layer of the old network, i.e., narrowing the differences of ${\vec{h^{\prime }}}_{i_{k}+1}\left(x\right)$ in the new network and ${\vec{h}}_{m_{k}}\left(x\right)$ in the old network based on the same input ${\vec{h}}_{m_{k}-1}\left(x\right)$.

Ideally, if we have an operator ◦ representing the mapping relationship, as shown in Equation (8) and Equation (9),


(8) $$\begin{array}{l} {\vec{h^{\prime }}}_{i_{k}+1}\left(x\right)=\left(W_{i_{k}}^{{\;}^{\prime }-},W_{i_{k}}^{{\;}^{\prime }+}\right)◦{\vec{h}}_{m_{k}-1}\left(x\right), \end{array}$$

(9) $$\begin{array}{l} {\vec{h}}_{m_{k}}\left(x\right)=W_{m_{k}}◦{\vec{h}}_{m_{k}-1}\left(x\right), \end{array}$$

and there exists a unique inverse operation of ◦, then we can easily reach the analytical solution of $\left(W_{i_{k}}^{{\;}^{\prime }-},W_{i_{k}}^{{\;}^{\prime }+}\right)$ as shown in Equation (10)


(10) $$\begin{array}{l} \left(W_{i_{k}}^{{\;}^{\prime }-},W_{i_{k}}^{{\;}^{\prime }+}\right)={\vec{h}}_{m_{k}}\left(x\right)◦inv\left({\vec{h}}_{m_{k}-1}\left(x\right)\right), \end{array}$$

where *inv*(·) represents the inverse operation. However, for networks with various operators and activation functions, the perfect situation is hard to realize. In the following, we will discuss how to provide a proper solution. In the following subsection, we will discuss two kinds of local network structures and provide proper solutions correspondingly.

### 3.2 Optimization for stage I

#### 3.2.1 Fully-connected network

For a fully-connected network function, the relationship in Equation (4) can be written as Equation (11),


(11) $$\begin{array}{l} {\vec{h}}_{k}\left(x\right)=\sigma \left(W_{k}{\vec{h}}_{k-1}\left(x\right)\right),\;k=1,2,\cdots \;,L. \end{array}$$

For simplicity, ${\vec{h}}_{k-1}\left(x\right)$ is replaced by *X* in the following part of this subsection. The solution of new inserted parameters can be obtained by solving Equation (12),


(12) $$\begin{array}{l} \underset{W_{i_{k}}^{{\;}^{\prime }-},W_{i_{k}}^{{\;}^{\prime }+}}{\min}∥W_{m_{k}}X-W_{i_{k}}^{{\;}^{\prime }+}\sigma \left(W_{i_{k}}^{{\;}^{\prime }-}X\right){∥}^{2}. \end{array}$$

in which, σ(·) is ReLU function,


$$ReLU(x)=\{\begin{array}{ccc} 0 & x & <0\\ x & x & \geq 0. \end{array}$$

It is a piecewise function and is irreversible. Therefore, if σ(·) in the optimization problem (12) is ReLU, it is unrealistic to derive an closed form solution. For providing an analytical solution most possibly, we can utilize its property of sparsity.

Considering $W_{i_{k}}^{{\;}^{\prime }+}\sigma \left(W_{i_{k}}^{{\;}^{\prime }-}X\right)$ in (12), we notice that $\sigma \left(W_{i_{k}}^{{\;}^{\prime }-}X\right)$ is sparse. Equation (12) can be regarded as a sparse coding problem as shown in Equation (13),


(13) $$\begin{array}{l} S=\overset{T}{\underset{t=1}{\sum }}z_{t}a_{t}=Bz, \end{array}$$

in which, *S* is the signal to be recovered, $B=\left[{\vec{b}}_{1},{\vec{b}}_{2},...,{\vec{b}}_{t}\right]$ are a group of basis vectors, and *z* = [*z*_1_, *z*_2_, ..., *z*_*M*_] are corresponding coefficients. For the optimization (12), *W*_*m*_*k*__*X*, $W_{i_{k}}^{{\;}^{\prime }+}$ and $\sigma \left(W_{i_{k}}^{{\;}^{\prime }-}X\right)$ can correspond to *S*, *B* and *z* in Equation (13), respectively. By setting $\Sigma_{W}=\sigma \left(W_{i_{k}}^{{\;}^{\prime }-}X\right)$, we have following optimization problem as shown in Equation (14),


(14) $$\begin{array}{l} \begin{array}{cc} \left(W_{i_{k}}^{{\;}^{\prime }+*},\Sigma_{w}^{*}\right)=\arg & \min∥W_{m_{k}}X-W_{i_{k}}^{{\;}^{\prime }+}\Sigma_{w}{∥}^{2}+\eta ∥\Sigma_{w}{∥}_{1}\\ s.t.\Sigma_{w}\geq 0, \end{array} \end{array}$$

in which, η is a hyperparameter. This optimization problem can be solved by alternately calculating $W_{i_{k}}^{{\;}^{\prime }+}$ and Σ_*w*_ via an KSVD method (Bertin et al., 2007; Farouk and Khalil, 2012) with non-negative matrix factorization.

$W_{i_{k}}^{{\;}^{\prime }-*}$ then can be obtained by solving the following problem, as shown in Equation (15),


(15) $$\begin{array}{l} W_{i_{k}}^{{\;}^{\prime }-*}=\arg\min∥M⊙\left(\overset{*}{\underset{W}{\Sigma }}-\left(W_{i_{k}}^{{\;}^{\prime }-}X\right)\right){∥}^{2}. \end{array}$$

where ⊙ is Hadamard product, and *M* is a index vector satisfying


$$\{\begin{array}{c} M_{j}=0\;if\;{\left(\Sigma_{W}^{*}\right)}_{j}=0\\ M_{j}=1\;if\;{\left(\Sigma_{W}^{*}\right)}_{j}>0. \end{array}$$

The verification experiments of this part are shown in Section 4.2.

#### 3.2.2 Convolution network

As the convolution operation makes tons of local computation and can be regarded as an severely sparse situation of fully-connection, it is too redundant to adopt the upmentioned algorithm. So, we use the chain derivative rule based on back propagation for solving the problem of convolution network.

To maintain generality, we adopt a functional representation for the multilayer neural network. In the functional representation, we view the neural network as a collection of mapping functions, with each function corresponding to a layer in the network. Assuming that we have a multi-layer neural network with *L*+1 layers. Each layer is represented by *h*_*i*_, where i ranges from 0 to *L*. We can define a functional ${\vec{h}}_{L}$ as follows. Suppose that the original shallower net is trained and expressed as Equation (16), which is a variant of Equation (3):


(16) $$\begin{array}{l} {\vec{h}}_{L}\left(x\right)=h_{L}◦h_{L-1}◦\cdots ◦h_{0}\left(x\right), \end{array}$$

where ◦ represents the composition operator for functions and ${\vec{h}}_{L}\left(x\right)$ denotes the output of the *L*th layer. Each module or component is denoted as *h*. *x* is the input of the network, and the dimension of *x* is adjusted according to the batch size of the dataset.

The matter is how to expand the original shallower net to the new deeper net. We note the structural similarity between the shallower and the new deeper nets. To make better use of the trained network, a new depth incremental learning model against the traditional end-to-end training model is proposed. The method splits any component *h*_*i*_ in ${\vec{h}}_{L}\left(x\right)$ as shown in Equation (17),


(17) $$\begin{array}{l} h_{i}\left(z\right)=f_{i}◦g_{i}\left(z\right), \end{array}$$

where z is the output of the functional before the i-th layer, which is represented as shown in Equation (18)


(18) $$\begin{array}{l} z=h_{i-1}◦\cdots ◦h_{0}\left(x\right), \end{array}$$

Then the optimization problem in Equation (6) is reformulated as follows:


(19) $$\begin{array}{l} \min∥h_{i}\left(z\right)-f_{i}◦g_{i}\left(z\right){∥}_{2}. \end{array}$$

According to Equation (19), the loss function is defined in Equation (20).


(20) $$\begin{array}{l} Loss=MSE\left(h_{i}\left(z\right)-f_{i}◦g_{i}\left(z\right)\right). \end{array}$$

The parameters of *f*_*i*_ and *g*_*i*_ are updated according to the loss function to approximate the optimization objective in Equation (19):

## 4 Experiment

We conduct more experiments on CNNs for their extensive use, in terms of convergence, effectiveness, and exploration.

### 4.1 Experiment setup

We evaluate our proposed DILS on object classification tasks with the following configurations. (1) ResNet-20, ResNet-32, and ResNet-44 (He et al., 2016) on CIFAR-10 (Krizhevsky and Hinton, 2009) are used to test the convergence and the accuracy improvement of the approach. (2) VGGNets (Simonyan and Zisserman, 2014) on CIFAR-10 and CIFAR-100 are used to test the accuracy and efficiency improvements of the approach. (3) ResNet-18 and ResNet-34 (He et al., 2016) on ImageNet (Deng et al., 2009) are used to visualize the training process of end-to-end and depth incremental learning strategies.

In the following experiments, ResNet20-32 is used to represent that ResNet-20 is extended to ResNet-32 by inserting layers, similar to VGGNet16-19. All the experiments are implemented with PyTorch (Paszke et al., 2019) on an NVIDIA GeForce RTX 3090 Ti GPU and two Intel(R) Xeon(R) Gold 6240 CPUs @ 2.60 GHz.

### 4.2 Experiment on fully connected network

To verify the effectiveness of sparse coding with dictionary learning in dealing with Stage I local stability-based supervision, the fitting experiment of function $y=x_{1}^{5}+x_{2}^{3}+x_{3}^{2}+x_{4}^{2}+x_{5}^{2}+x_{6}+x_{7}+x_{8}+x_{9}+x_{10}$ is conducted. In total, 3,000 points are sampled uniformly from value −1 to 1 for the training set, and 200 points are sampled uniformly from value −1 to 1 for the test set. A four-layer fully connected neural network activated by ReLU is used to fit the function.

The results are shown in Table 1. DILS on fully connected network performs better than end-to-end training model for the robustness of Stage I local stability supervision. The reason is that sparse coding is another manifestation of depth incremental learning model, so the loss is closed to depth incremental learning model. However, sparse encoding does not provide the same excellent stability supervision as depth incremental learning model, so the results are slightly inferior.

**Table 1:** Sparse coding with dictionary learning experiment results on the fully connected network.

**Training strategy**	**Iterations**	**Training loss**	**Test loss**
End-to-end	4,000	2.96e-2	3.10e-2
	7,000	1.22e-2	1.29e-2
	12,000	6.85e-5	1.28e-4
DILS	4,000	9.13e-4	1.16e-3
	7,000	9.88e-5	1.11e-3
	12,000	3.55e-5	8.76e-5

We adopt end-to-end and DILS training for 4,000, 7,000, 12,000 iterations, respectively, and report the training loss and test loss.

### 4.3 Convergence validation of DILS

To demonstrate the convergence of DILS, we devised the following two experiments to test the convergence of the training process and results. The training process convergence experiment is performed on VGGnet13-16, while the experimental results convergence experiment is performed on ResNet20-32-44. Both experiments are performed on CIFAR-10.

**Figure 4:**
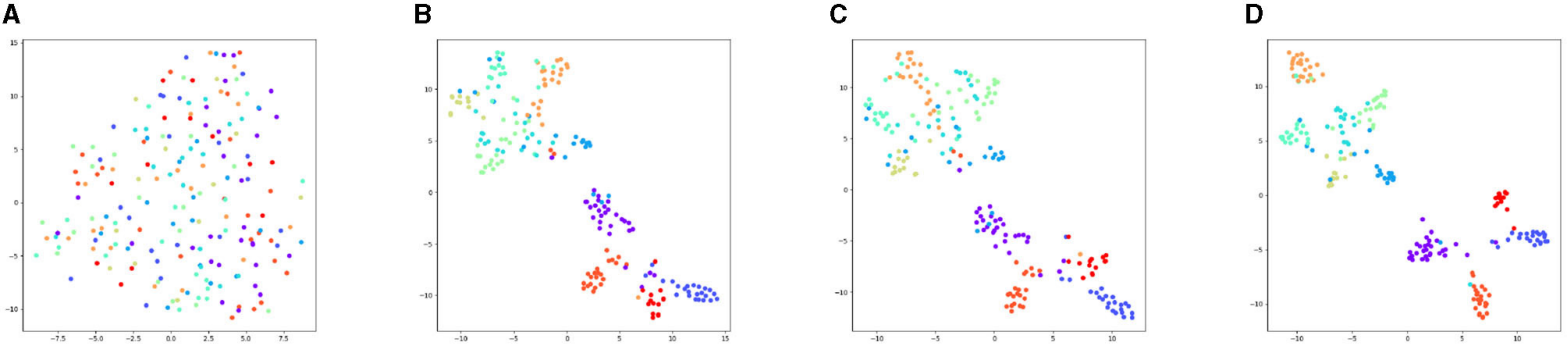
Feature convergence graph of training process of VGGnet13-16 on CIFAR-10. **(A)** The data points of each category are scattered due to random initialization. **(B)** After training VGGNet-13 for 20 epochs, the distribution of the features of the network shows a tendency to converge. **(C)** The Stage I local supervision ensures network stability, so that the figure is similar to the trained VGGNet-13 shown in **(B)**. **(D)** After the stage II global supervision, which guaranteeing the network plasticity, the category distribution is more concentrated. The intra-class feature distance shrinks and the inter-class feature distance increases, which leads to better classification results.

Figure 4 depicts the feature convergence process of DILS, using 200 samples on VGGNet13-16 with CIFAR-10. First, a randomly initialized network is shown in Figure 4A. Then we train 20 epochs on the VGGNet-13, which is shown in Figure 4B. After that, localized supervision is applied to the new growth layer, which can guarantee the stability of the network, as shown in Figure 4C. Finally, global supervision is imposed on the entire network, exploiting the plasticity of the network to achieve better classification results. The finally result is shown in Figure 4D.

#### 4.3.1 Experiment results

To validate that the experimental results do not diverge with the increase of the inserted layers, the ResNet20-32-44 experiments on CIFAR-10 are established. The experimental results are shown in Table 2. By using DILS, the network depth increases from ResNet-20 to ResNet-32, further increasing to ResNet-44. The network accuracy steadily improves as the network depth increases. Due to the Stage I local stability supervision, the network shows no sudden drop in accuracy and benefits from the Stage II global plasticity supervision, and the result is improved to some extent.

**Table 2:** Convergence validation of DILS.

**Strategy**	**ResNet20**	**ResNet32**	**ResNet44**
BL acc. (%)	91.25	92.49	92.83
Ours acc. (%)	91.29	92.53	92.8

“BL” denotes the baseline network. The results of ResNet20-32-44 by DILS converges similarly as the commonly trained ResNet20, ResNet32, and ResNet44.

### 4.4 Comparison of end-to-end learning strategy and DILS

#### 4.4.1 Training process

**Figure 5:**
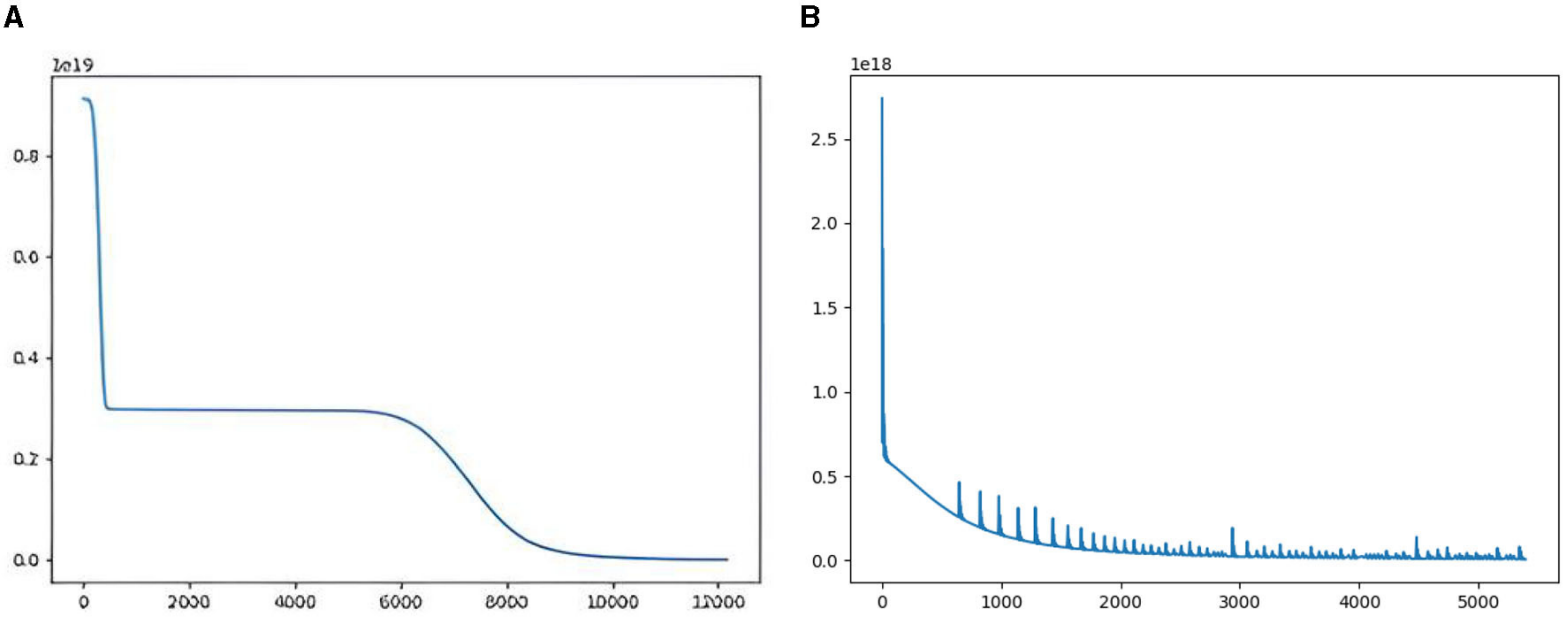
Comparison of end-to-end learning strategy and DILS. **(A)** Shows end-to-end learning strategy, where the loss falls in a stepwise manner. **(B)** Shows DILS, where the loss drops continuously and converges faster.

**Fully connected networks with function fitting:** To compare the differences between the two learning strategies on training process, we fit $y=x_{1}^{5}+x_{2}^{3}+x_{3}^{2}+x_{4}^{2}+x_{5}^{2}+x_{6}+x_{7}+x_{8}+x_{9}+x_{10}$ with a four-layer fully connected network. Three-thousand points are sampled uniformly from value −100 to 100 for the training set. The training characteristics of the two learning strategies can be seen from the loss descent curve. In Figure 5A, the loss curve descends in steps for taking different learning rates 0.005 and 0.0005, while in Figure 5B, the curve drops continuously and converges faster with fixed learning rate 0.005. In the end-to-end learning strategy, the network converges after 12,000 iterations. However, in the depth incremental learning strategy, the same network converges after 3,000 iterations.

#### 4.4.2 Experiment results

In order to prove the effectiveness of depth incremental learning strategy, two kinds of experiments based on VGGNet13-16 and ResNet20-32 network are established. In the VGGNet13-16 experiment, the number of epochs in end to end training model and depth incremental learning model are equal. In the ResNet20-32 experiment, the calculation amounts of both models are equal.

**VGGnet with CIFAR-10 and CIFAR-100:** To prove the effectiveness of DILS, an experiment based on VGGNet13-16 is established. The number of epochs in the end-to-end training model and depth incremental learning model are equal. The experimental accuracy and number of parameters quantity are compared. Two training models are trained in 30, 70, and 100 epochs on CIFAR-10 and CIFAR-100, respectively. The end-to-end training model is trained directly on VGGNet-16, and the depth incremental learning model is divided into three steps. The first step is training the original shallow VGGNet-13. The second step is Stage I local stability-based supervision. The third step is Stage II global plasticity-based supervision. The total epochs of the three steps are equal to the epochs of end-to-end training. The experiments are shown in Tables 3, 4. Table 5 shows parameters comparison of two learning strategies on VGGNet on CIFAR-100. The experiment shows that the training parameters decrease by 6.96*x*10^3^ MB and the accuracy increases by 3.08%.

**Table 3:** Accuracy comparison of the two training strategies on CIFAR-100.

**Network**	**Epoch**	**Accuracy**
VGGNet-16	30	59.22%
	70	63.24%
	100	64.55%
VGGNet13-16 (ours)	10 + 10 + 10	60.40%
	30 + 10 + 30	65.90%
	60 + 30 + 10	67.63%

“60 + 30 + 10” denotes that the shallow prior network is trained for 60 epochs, and Stage I local supervision is trained for 30 epochs, and Stage II global supervision is trained for 10 epochs. The following tables use the same notation. The results show that within the same total training epochs, VGGNet13-16 by our DILS achieves better accuracy than baseline VGGNet-16.

**Table 4:** Accuracy comparison of the two training strategies on CIFAR-10.

**Network**	**Epoch**	**Accuracy**
VGGNet-16	30	87.58%
	70	89.83%
	100	90.05%
VGGNet13-16 (ours)	10 + 10 + 10	87.92%
	30 + 10 + 30	90.10%
	60 + 30 + 10	91.23%

The results show that within the same total training epochs, VGGNet13-16 by our DILS achieves better accuracy than baseline VGGNet-16.

**Table 5:** The comparison of the number of parameters of the two learning strategy.

**Networks**		**Epoch**	**Params↓** **(× 10^3^ MB)**	**Accuracy↑** **(%)**
VGGNet-16		100	135.12	64.55
VGGNet13-16 (ours)	VGGNet-13	60	81.07	–
	Stage I	10	12.82	–
	Stage II	30	34.27	–
	Total	100	128.16 (6.96↓)	67.63 (3.08↑)

We conduct the experiment of VGGNet-16 and VGGNet13-16 by DILS on CIFAR-100. The results show that within the same total training epochs, VGGNet13-16 by DILS achieves 3.08% better accuracy while 6.96 M less parameters. They are the value of 135.12–128.16 and 67.63–64.55, respectively and represent the decreasing value of the parameters quantity and the rising value of the accuracy.

**Figure 6:**
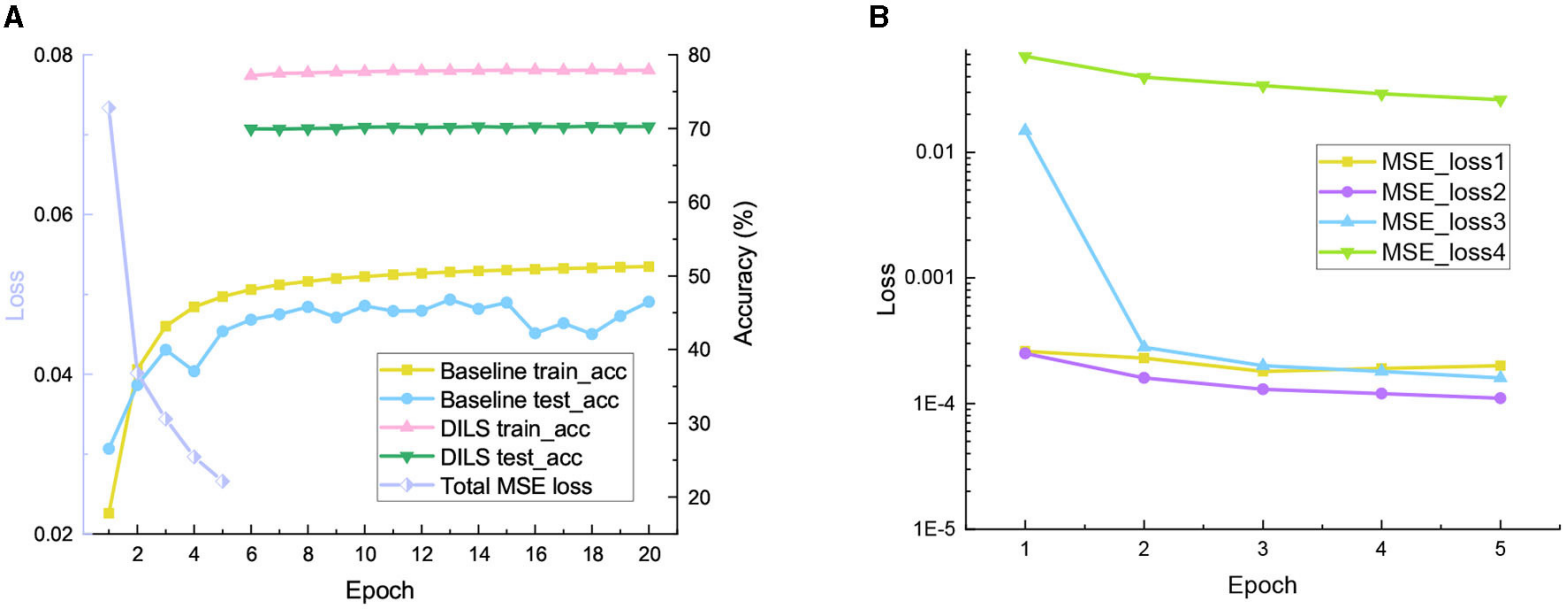
Experimental results on ResNet with ImageNet dataset. **(A)** Train accuracy, test accuracy, and total MSE loss. **(B)** The MSE loss for four positions in local stability supervision.

**ResNet with ImageNet:** To further observe the training process of DILS, experiments are conducted on the ResNet18-34 network with the ImageNet dataset. The pretrained ResNet18 network is used. Original shallow network, and in the first stage, local stability supervision is conducted for five epochs using MSE as the loss function, with a learning rate of 0.005. In the second stage, global plasticity supervision is conducted for 30 epochs with a learning rate of 1e-5. In fact, 10 epochs are sufficient to ensure the convergence of the DILS network. To facilitate comparison with the naive end-to-end network, we set the total number of epochs to 35. The learning rate of the plain end-to-end network is initially set to 0.1 and multiplied by 0.1 every 30 epochs. The experimental results are shown in the Figure 6. The experimental results demonstrate that the network trained using the DILS strategy can achieve faster convergence due to its ability to inherit prior knowledge from the existing network. Additionally, an interesting observation is that the MSE loss of the front layers in the network is relatively small, while the MSE loss increases as it gets closer to the back layers, as shown in Figure 6B. This may be because the deeper layers of the network contain richer semantic information, which is more important for the representation of the classification network.

It is worth mentioning that the training model we proposed uses a single learning rate at each step, while the end-to-end training model uses multiple variable learning rates to obtain the best experimental results. The proposed model effectively alleviates the problem of learning rate adjustment in DNN training and obtains better experimental results.

### 4.5 Exploration of the rule on the DILS

**Figure 7:**
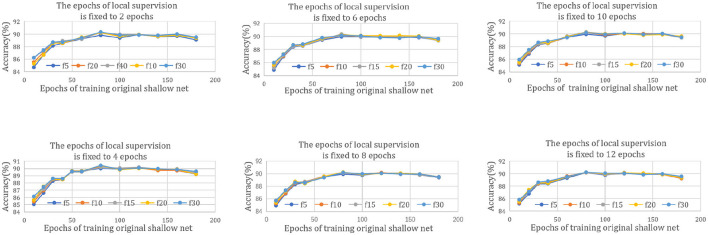
Rule of DILS on ResNet20-32. The six subfigures represent different epochs taken for the stage I local stability-based supervision, 2, 4, 6, 8, 10, and 12 epochs, respectively. For every subfigure, chart abscissa represents the number of epochs used for training the original shallow net and different curves correspond to different stage I global supervision epochs.

To obtain better experimental results, we try to explore the general rule of DILS. A series of experiments are carried out in ResNet20-32. In Figure 7, different subfigures show different epochs in which Stage I is trained. For example, in the first subfigure of Figure 7, the I local supervision epochs of all curves are fixed to 2, and the rest of the subfigures are fixed to 4, 6, 8, 10, and 12 epochs in order. The chart abscissa represents the number of epochs training the original shallow net. Different color curves correspond to different epochs of Stage II. For example, “f5” in the legend indicates five epochs of the II global supervision. The optimal training number of the original shallow network is fixed at 80, regardless of how many epochs the local and the global supervisions are trained.

The reason for this phenomenon is that too few iterations cannot guarantee net stability, and too many iterations limit the plasticity of the net. Experiments show that the most plausible prior network exists. This reflects the scientificity of the proposed strategy.

### 4.6 The reasonability of the stage I

The proposed method makes use of the priors of the shallow network and it is extended to the deep network by fitting the original one layer with multiple layers. To validate its reasonability, an experiment is performed by randomly initializing the parameters of any layer in the well-trained VGGNet-16 on CIFAR-10, as shown in Table 6. Then, by extracting the output before and after the random initialized layer, DILS is applied to this layer. In the experiment, one layer and two layers are used to fit the original layer, respectively. As the number of training epochs increases, the accuracy improves. In addition, the results of “two-layer” are better than those of “one-layer”.

**Table 6:** The results of using one or two layers to fit one layer.

**Training strategy**	**One-layer acc**.	**Two-layer acc**.
Baseline	90.05%	90.05%
R/N	10%	10%
5 epoch	81.55%	82.27%
10 epoch	81.97%	88.93%
15 epoch	82.96%	89.28%
20 epoch	83.26%	89.18%

“R/N” denotes random initialization. In the “one-layer” column, we fit one layer in the original network with one layer. In the “two-layer” column, we fit one layer in the original net with two layers. As the number of training epochs grows, the gradually converging results validate the reasonability of Stage I.

### 4.7 The exploration of the training mode in stage I

To explore the best training mode to train the inserted layer in the Stage I local supervision, four experiments are conducted on VGGNet16-17 with complete training and incomplete training prior nets. Two training modes are compared. One is the end-to-end training mode, which means taking the input of the network as input and taking the output of the network as output. The other uses the input and output before and after the inserted-layers as the input and output of the local supervision. Experiments are performed with CIFAR-10. According to Table 7, with a complete VGGNet-16 trained for 80 epochs, the depth incremental learning mode achieves better experimental results, while with the incomplete VGGNet-16 trained for 10 epochs, the end-to-end training mode achieves better experimental results.

**Table 7:** Comparison of two training modes in stage I on CIFAR 10.

**Training stage**	**VGGNet-16**	**Stage I**	**Stage II**
**Depth incremental learning mode in stage. I**			
Incomplete acc.(%)	86.69	86.62	87.87
Complete acc.(%)	90.68	90.41	91.56
**End-to-end training mode in stage. I**			
Incomplete acc.(%)	86.69	87.58	88.41
Complete acc.(%)	90.68	90.02	91.15

The reason is summarized as follows. In a well-trained original shallow net, the feasible region of the solution is better, so that DILS can find the approximate optimal solution in a smaller feasible region with a finite number of epochs. However, with the incomplete training mode, the original shallow network does not provide a good feasible region, so the end-to-end training mode benefits from a large feasible region.

## 5 Conclusion

This study proposes a depth incremental learning strategy (DILS), which explores the importance of knowledge transfer in the field of neural networks, with a specific focus on transferring knowledge from small networks to large networks. In the procedure, the new inserted layers are derived by the existing network, so that they can best inherit previously learned knowledge. It not only enhances the training efficiency, more importantly, it provides a more flexible way to obtain a series of networks with various complexities and corresponding accuracies. It makes the network have better extensibility and have the potential to be utilized for more complex network design in the future.

## Data availability statement

The original contributions presented in the study are included in the article/supplementary material, further inquiries can be directed to the corresponding author.

## Author contributions

YW: Data curation, Methodology, Software, Validation, Visualization, Writing – original draft. ZH: Funding acquisition, Investigation, Methodology, Resources, Writing – original draft. SY: Validation, Writing – review & editing. SZ: Formal analysis, Writing – review & editing. BL: Software, Writing – review & editing. HF: Funding acquisition, Writing – review & editing.
